# The mediating effect of emotion dysregulation on the relationship between childhood maltreatment and suicidal ideation in patients with depression

**DOI:** 10.3389/fpsyg.2026.1776388

**Published:** 2026-03-16

**Authors:** Lina Chen, Yuling Feng, Yong Wang, Nana Xiong, Lixia Li, Xia Li

**Affiliations:** 1 School of Medicine, Jiangsu University, Zhenjiang, China; 2 Peking University Sixth Hospital, Beijing, China; 3 Intravenous Infusion Therapy Center, Zhenjiang First People’s Hospital, Zhenjiang, China

**Keywords:** childhood maltreatment, depression, emotion dysregulation, mediating effect, suicidal ideation

## Abstract

**Background:**

Childhood maltreatment is a well-established risk factor for suicidal ideation in patients with depression. However, the psychological mechanisms linking early adversity to current suicide risk remain incompletely understood. Early traumatic experiences may impair emotion regulation capacities, thereby exacerbating suicidal ideation.

**Objective:**

This study aimed to clarify the association between childhood maltreatment and suicidal ideation in depression by examining the potential mediating role of emotion regulation difficulties.

**Methods:**

We recruited 595 inpatients meeting ICD-10 criteria for a depressive episode. Participants underwent a comprehensive assessment, including sociodemographic and clinical data collection. Childhood maltreatment, suicidal ideation, and emotion regulation difficulties were evaluated using the Childhood Trauma Questionnaire (CTQ), the Beck Scale for Suicide Ideation-Chinese Version (BSI-CV), and the Difficulties in Emotion Regulation Scale (DERS), respectively. Pearson correlation analysis was used to examine associations among childhood maltreatment, difficulties in emotion regulation, and suicidal ideation. Structural equation modeling (SEM) and the bootstrap method were used to test the mediating effect, and the model was modified transparently according to modification indices.

**Results:**

Childhood maltreatment was significantly associated with higher levels of suicidal ideation in patients with depression. Furthermore, greater difficulties in emotion regulation were linked to increased suicidal ideation. Critically, mediation analysis indicated that emotion regulation difficulties partially mediated the relationship between childhood maltreatment and suicidal ideation.

**Conclusion:**

These findings suggest that childhood maltreatment exerts both a direct effect on suicidal ideation in patients with depression and an indirect effect through the exacerbation of difficulties in emotion regulation.

## 1 Introduction

Suicide is a major public health problem worldwide. According to data from the World Health Organization (WHO), approximately 720,000 people die by suicide each year, and suicide has become the third leading cause of death among individuals aged 15 to 29 years (Wang et al., 2014; Jiang et al., 2020). Suicidal ideation is regarded as the most reliable predictor of suicidal behavior (Franklin et al., 2017). Among patients with depression, suicidal ideation has particularly important clinical implications. Epidemiological studies indicate that approximately 70% of depression patients experience suicidal ideation, that more than one million deaths by suicide each year are attributable to depression, and that suicide risk increases in a dose dependent manner with the duration and severity of depressive episodes (Klonsky et al., 2016; Ladi-Akinyemi et al., 2023). As a critical juncture in the pathway to suicide, elucidating the risk factors for suicidal ideation and the mechanisms by which they operate is of paramount practical importance.

Existing research has demonstrated a robust association between childhood maltreatment and suicidal ideation, and individuals with a history of childhood maltreatment show heightened vulnerability to depressive symptoms and suicidal thoughts (Lin et al., 2020; Wang et al., 2022). (O’Connor et al., 2011; Turecki and Brent, 2016; O’Connor and Kirtley, 2018) proposed the Integrated Motivational and Volitional model of suicidal behavior, which conceptualizes childhood trauma as a pre-motivational risk factor. Such trauma may influence the epigenetic regulation of genes and alter cortisol regulation, thereby contributing to the emergence of suicidal ideation. Longitudinal studies have shown that childhood maltreatment may increase the risk of depression and suicide in adulthood through mechanisms such as impaired emotion regulation and the reinforcement of negative cognitive patterns (Giano et al., 2020). However, findings across studies are not entirely consistent. Some work emphasizes a strong association between emotional abuse and suicidal ideation (de Araújo and Lara, 2016), whereas other studies report that the effects of physical abuse or neglect are not significant (Berardelli et al., 2022). Patients with depression who have experienced childhood maltreatment often show a higher propensity toward suicide, and the persistent presence of suicidal ideation further increases suicide risk and subjective distress. Nevertheless, the association between childhood maltreatment and suicidal ideation specifically among patients with depression has not been fully clarified. This heterogeneity underscores the need for more nuanced analyses of the distinct dimensions of childhood maltreatment, taking cultural context into account.

Emotion Dysregulation have been identified as a transdiagnostic risk factor for adolescent psychopathology (Nock, 2010; Gratz and Roemer, 2004). This construct refers to deficits in the ability to identify, accept and modulate emotional states, and suicidal ideation is one of the psychopathological outcomes associated with such difficulties. A large body of theoretical (Law et al., 2015; Heffer and Willoughby, 2018) and empirical research (Miller et al., 2018; Brausch and Woods, 2019) indicates that both general emotion dysregulation and specific emotion regulation problems are linked to increased risk of suicidal ideation. Individuals who experience pronounced emotion dysregulation (Law et al., 2015) are more likely to develop feelings of thwarted belongingness, hopelessness and other psychological states that heighten the likelihood of suicidal ideation, and when cumulative distress reaches a sufficiently high level, the risk of suicidal behavior is further strengthened. Theoretical models propose that childhood maltreatment may disrupt the development of adaptive emotion regulation strategies, leading to a chronically dysregulated emotional state that amplifies suicidal ideation (Teisl and Cicchetti, 2008; Berzenski, 2019). However, most existing studies have focused on the association between Emotion Dysregulation and symptoms of depression and anxiety. The mediating role of Emotion Dysregulation in the relationship between childhood maltreatment and suicidal ideation has not been systematically tested, and evidence is particularly scarce in clinical samples of patients with depression (Demir et al., 2020; Khosravani et al., 2021). Thus, the interplay among childhood maltreatment, difficulties in emotion regulation, and suicidal ideation warrants further investigation.

On this basis, the present study focused on inpatients with depression and aimed to: (1) assess childhood maltreatment, suicidal ideation and difficulties in emotion regulation, and examine the associations between different dimensions of childhood maltreatment and suicidal ideation; and (2) test the mediating role of difficulties in emotion regulation. The findings are expected to provide a theoretical foundation for suicide risk interventions in patients with depression and to offer empirical support for psychotherapeutic strategies that target emotion regulation.

## 2 Methods

### 2.1 Participants

This cross-sectional study determined its sample size using the formula for cross-sectional surveys presented in the 5th edition of Medical Statistics: N=Zα22P(1−P)δ2 . According to previous research Kim et al., (2021), the detection rate (prevalence) of suicidal ideation among depression patients is 70%,thus P =0.7, The type I error rate was set at α = 0.05, givingZα2 =1.96, and the allowable margin of error was δ =0.04. Based on these parameters, the required sample size was calculated to be 504. Allowing for an anticipated attrition rate of 15%, a minimum of 580 participants was needed. Subsequently, inpatients with depression were recruited from a tertiary psychiatric hospital in Beijing.

Inclusion criteria were as follows: (1) meeting the diagnostic criteria for depressive disorder in the 10th revision of the International Classification of Diseases (ICD-10); (2) diagnosis confirmed independently by at least two psychiatrists with an attending or higher professional title; (3) age between 16 and 35 years, with no restriction on sex; and (4) sufficient capacity to complete all assessments and to fully understand the content of the informed consent form.

Exclusion criteria were: (1) presence of organic mental disorders of cerebral origin; (2) substance dependence; (3) intellectual disability, cognitive impairment or other major psychiatric disorders; and (4) severe physical illness.

The final valid sample comprised 595 participants, yielding a response rate of 96.12%. All participants provided written informed consent; for minors, written informed consent was also obtained from their legal guardians. The study protocol was approved by the Medical Ethics Committee of Peking University Sixth Hospital (approval No. 2023–44).

### 2.2 Measures

#### 2.2.1 General information questionnaire

A self-developed general information questionnaire was used to collect demographic and clinical characteristics, including age, sex, years of education, marital status, employment status, only-child status, monthly family income, living arrangements, age at first onset of depression, duration of illness, number of depressive episodes or relapses, smoking and alcohol use, and family history of psychiatric disorders.

#### 2.2.2 Childhood trauma questionnaire (CTQ)

Childhood maltreatment was assessed with the Childhood Trauma Questionnaire, which provides a comprehensive evaluation of adverse experiences in childhood (Zhao et al., 2005). The scale consists of 28 items and includes five subscales: emotional abuse, emotional neglect, physical abuse, physical neglect and sexual abuse. Each item is rated on a five-point scale ranging from 1 (“never”) to 5 (“always”). The average item score was calculated, with scores ranging from 1 to 5. Higher scores indicate more severe childhood maltreatment. In the present study, the Cronbach’s *α* coefficient for the total scale was 0.90. The Cronbach’s α coefficients for the emotional abuse, emotional neglect, physical abuse, physical neglect and sexual abuse subscales were 0.82, 0.88, 0.87, 0.61 and 0.82, respectively.

#### 2.2.3 Beck scale for suicide ideation—Chinese version (BSI-CV)

Suicidal ideation was measured using the Chinese version of the Beck Scale for Suicide Ideation (Li et al., 2011). This instrument assesses the severity of suicidal ideation during the past week and at the time when the respondent felt most depressed. It comprises two subscales, suicidal ideation and suicide risk, with a total of 19 items. Each item consists of three statements scored from 0 to 2. For example, the item “To what extent do you wish to live?” is scored as 0 (“moderate to strong wish to live”), 1 (“weak wish to live”) or 2 (“no wish to live”). A nonzero score on item 4 or item 5 indicates the presence of suicidal ideation, and higher scores reflect stronger suicidal ideation. Each question is asked for two time frames: the current week and the period when the respondent was most depressed. In the present study, the Cronbach’s *α* coefficient for the suicidal ideation subscale was 0.86.

#### 2.2.4 Difficulties in emotion regulation scale (DERS)

Emotion Dysregulation were assessed using the Difficulties in Emotion Regulation Scale, which evaluates an individual’s ability to use emotion regulation strategies effectively (Gratz and Roemer, 2004). The scale consists of 36 items and six subscales: difficulties in perceiving emotions, difficulties in identifying emotions, nonacceptance of emotional responses, difficulties in goal-directed behavior, impulse control difficulties and limited access to effective emotion regulation strategies. Items are rated on a five-point scale from 1 (“almost never”) to 5 (“almost always”). The average item score was calculated as an indicator of emotion dysregulation, with scores ranging from 1 to 5. Higher scores indicate greater difficulties in emotion regulation and poorer emotion regulation capacity. In this study, the Cronbach’s *α* coefficient for the total scale was 0.94.

### 2.3 Statistical analysis

Data were analyzed using SPSS 26.0 and Amos 28.0. Pearson correlation analyses were conducted to examine the relationships among childhood maltreatment, difficulties in emotion regulation, and suicidal ideation. To ensure score comparability across different scales and to facilitate the interpretation of path coefficients, all variables were calculated as item means for statistical analysis.

A structural equation model (SEM) was constructed using Amos 28.0, with childhood maltreatment specified as the independent variable, difficulties in emotion regulation as the mediating variable, and suicidal ideation as the dependent variable. To control for potential confounding effects of clinical and demographic characteristics, sex, family history, educational level, age at first onset, illness duration, and number of episodes/relapses were included as control variables in the model. Parameters were estimated using the maximum likelihood method, and the significance of the mediating effect was tested using the bootstrap method with 2,000 resamples. Effects were considered statistically significant if the 95% bias-corrected confidence interval for the path coefficient or mediating effect did not contain zero.

## 3 Results

### 3.1 Correlational analysis between childhood maltreatment and suicidal ideation

Correlations between childhood maltreatment (total score and subdimensions) and suicidal ideation were examined, as shown in Table 1. In this study, to eliminate the influence of varying item numbers across different scales on total score weighting, all variables were calculated as item means for statistical analysis. Suicidal ideation scores were derived from the mean of BSI-CV items assessing suicidal ideation during the past week (BS1) and during the period when respondents felt most depressed (BS2). The use of item means not only provides an intuitive reflection of participants’ average score per item but also facilitates the comparison and interpretation of path coefficients in subsequent structural equation modeling. The results indicated that, with the exception of sexual abuse, childhood maltreatment and its subdimensions were significantly and positively associated with suicidal ideation. Specifically, childhood maltreatment was significantly and moderately positively correlated with suicidal ideation during the past week (*r* = 0.27, *p* < 0.01) and during the most depressed period (*r* = 0.31, *p* < 0.01). With regard to the subdimensions of childhood maltreatment, physical abuse (*r* = 0.16, *p* < 0.01), emotional abuse (*r* = 0.36, *p* < 0.01), physical neglect (*r* = 0.22, *p* < 0.01) and emotional neglect (*r* = 0.31, *p* < 0.01) were all significantly correlated with suicidal ideation. These correlational findings provide preliminary support for the study hypotheses and justify further analyses.

**Table 1:** Correlational analysis of childhood maltreatment and suicidal ideation.

Variable	1	2	3	4	5	6	7	8	9
1. Physical abuse									
2. Emotional abuse	0.52^**^	–							
3. Physical neglect	0.43^**^	0.50^**^	–						
4. Emotional neglect	0.41^**^	0.61^**^	0.72^**^	–					
5. Sexual abuse	0.18^**^	0.18^**^	0.13^**^	0.09^*^	–				
6. Childhood maltreatment	0.71^**^	0.84^**^	0.78^**^	0.84^**^	0.34^**^	–			
7. Suicidal ideation during the past week	0.12^**^	0.30^**^	0.20^**^	0.23^**^	0.03	0.27^**^	–		
8. Suicidal ideation during the most depressed period	0.17^**^	0.34^**^	0.19^**^	0.32^**^	0.02	0.31^**^	0.56^**^	–	
8. Suicidal ideation	0.16^**^	0.36^**^	0.22^**^	0.31^**^	0.03	0.33^**^	0.91^**^	0.86^**^	
M	1.64	2.52	1.74	2.77	1.22	1.78	1.65	2.42	2.04
SD	0.86	1.19	0.77	1.15	0.56	0.60	0.81	0.66	0.65

**denotes: significance.

### 3.2 Correlational analysis between difficulties in emotion regulation and suicidal ideation

Correlations between difficulties in emotion regulation (total score and subdimensions) and suicidal ideation were examined, as shown in Table 2. Similarly, difficulties in emotion regulation and its subdimensions were calculated as item means to ensure logical consistency in score ranges with suicidal ideation. The results indicated that difficulties in emotion regulation and its subdimensions were significantly and positively correlated with suicidal ideation and its subdimensions. Specifically, difficulties in emotion regulation were significantly and strongly positively correlated with suicidal ideation during the past week (*r* = 0.50, *p* < 0.01) and during the most depressed period (*r* = 0.48, *p* < 0.01). With regard to the subdimensions of difficulties in emotion regulation, nonacceptance of emotional responses (*r* = 0.30, *p* < 0.01), difficulties in perceiving emotions (*r* = 0.25, p < 0.01), limited access to effective emotion regulation strategies (*r* = 0.48, *p* < 0.01), impulse control difficulties (*r* = 0.48, *p* < 0.01), difficulties in identifying emotions (*r* = 0.40, *p* < 0.01) and difficulties in goal-directed behavior (*r* = 0.42, *p* < 0.01) were all significantly correlated with suicidal ideation. These correlational findings provide preliminary support for the study hypotheses and warrant further analysis.

**Table 2:** Correlational analysis of difficulties in emotion regulation and suicidal ideation.

Variable	1	2	3	4	5	6	7	8	9	10
1. Non-acceptance										
2. Goals	0.10									
3. Impulsivity	0.54^**^	0.11^**^								
4. Awareness	0.46^**^	0.16^**^	0.71^**^							
5. Strategy	0.30^**^	0.51^**^	0.42^**^	0.34^**^						
6. Clarity	0.42^**^	0.01	0.73^**^	0.67^**^	0.29^**^					
7. Emotion dysregulation	0.67^**^	0.39^**^	0.87^**^	0.82^**^	0.66^**^	0.74^**^				
8. Suicidal ideation during the past week	0.27^**^	0.23^**^	0.44^**^	0.43^**^	0.39^**^	0.34^**^	0.50^**^			
9. Suicidal ideation during the most depressed period	0.26^**^	0.21^**^	0.40^**^	0.43^**^	0.31^**^	0.41^**^	0.48^**^	0.56^**^		
10. Suicidal ideation	0.30^**^	0.25^**^	0.48^**^	0.48^**^	0.40^**^	0.42^**^	0.56^**^	0.91^**^	0.86^**^	
M	3.00	2.75	3.59	3.48	2.93	4.02	3.30	1.65	2.42	2.04
SD	1.07	0.87	0.94	1.02	1.01	0.85	0.68	0.81	0.66	0.65

**denotes: significance.

### 3.3 Mediating role of difficulties in emotion regulation between childhood maltreatment and suicidal ideation

**Figure 1:**
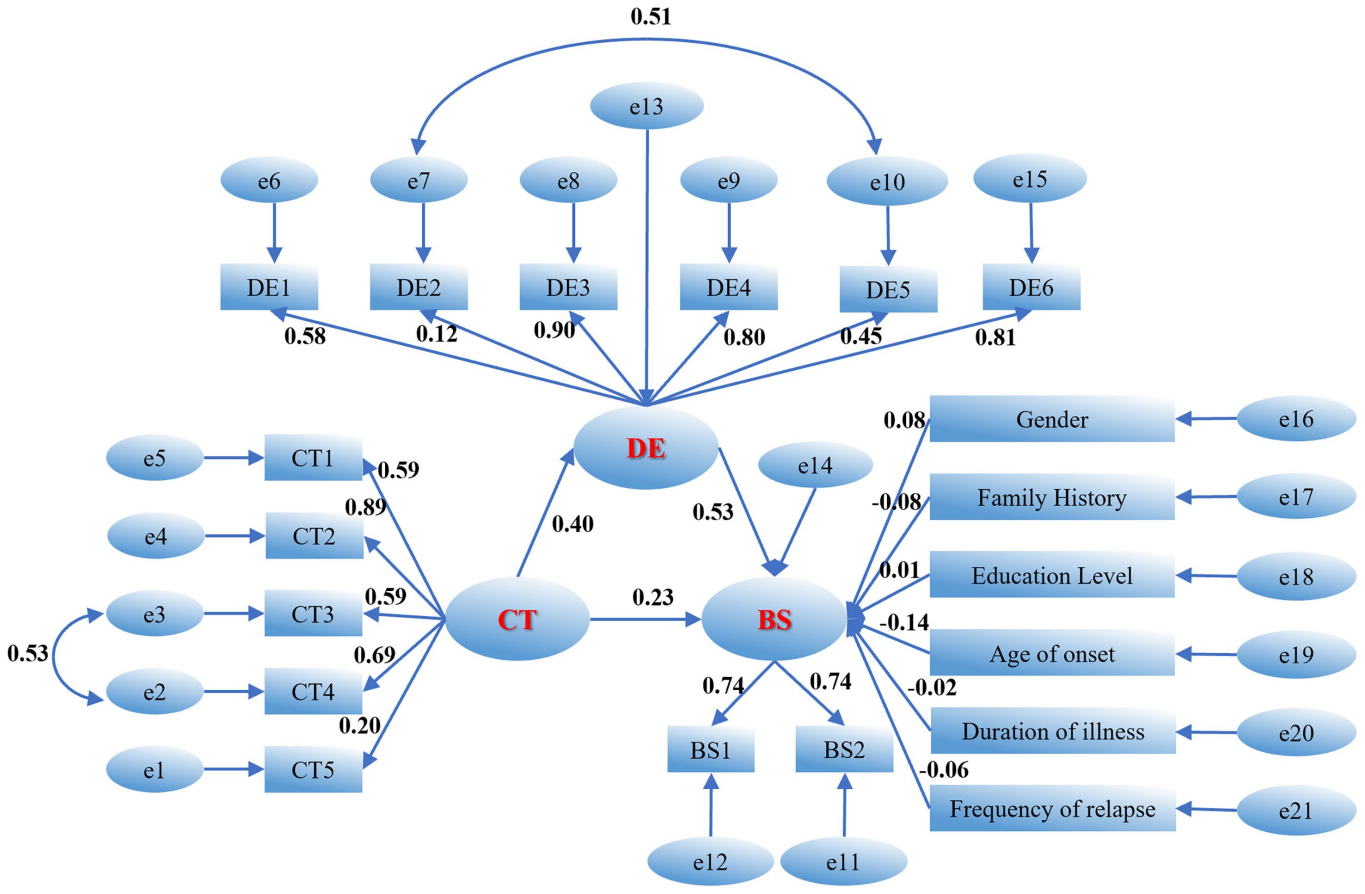
Mediation model of emotion dysregulation. *e*, error term, CT1, physical abuse; CT2, emotional abuse; CT3, physical neglect; CT4, emotional neglect; CT5, sexual abuse; DE1, non-acceptance; DE2, goals; DE3, impulsivity; DE4, awareness, DE5, strategy; DE6, clarity; BS1, suicidal ideation during the past week; BS2, suicidal ideation during the most depressed period.

Based on the correlational findings, childhood maltreatment and difficulties in emotion regulation were both positively correlated with suicidal ideation and its two subdimensions (suicidal ideation during the past week and during the most depressed period). Therefore, a structural equation model (SEM) was constructed using Amos 28.0, with childhood maltreatment (CT) specified as the independent variable, difficulties in emotion regulation (DE) as the mediating variable, and suicidal ideation (BS) as the dependent variable. Sex, family history, educational level, age at first onset, illness duration, and number of episodes/relapses were included as control variables (Figure 1). In the measurement model, CT1 to CT5 represented the five subdimensions of childhood maltreatment (physical abuse, emotional abuse, physical neglect, emotional neglect, and sexual abuse); DE1 to DE6 represented the six subdimensions of difficulties in emotion regulation (Non-acceptance, Goals, Impulsivity, Awareness, Strategy, Clarity); BS1 and BS2 assessed suicidal ideation during the past week and during the most depressed period, respectively. All scales were analyzed using item means.

Following the construction of the initial structural equation model, its fit was first examined. The initial model fit indices indicated that some parameters did not reach optimal levels: χ^2^/df = 7.942, CFI = 0.719, TLI = 0.678. To further optimize model quality, transparent modifications were made to the measurement model based on modification indices (MI) while adhering to theoretical logic. Specific modification paths included establishing a covariance between residuals e2 (emotional abuse) and e3 (physical neglect) under the childhood maltreatment latent variable, and between residuals e7 (Goals) and e10 (Strategy) under the difficulties in emotion regulation latent variable. The rationale for these modifications was that the correlated dimensions belonged to the same latent variable and exhibited some semantic overlap. For instance, difficulties in perceiving and identifying emotions both involve cognitive processing of emotional information; therefore, correlated measurement errors were considered statistically and theoretically justifiable.

Following these modifications, the model fit indices improved substantially, with χ^2^/df decreasing to 6.314, CFI increasing to 0.788, and TLI increasing to 0.753. Although some indices did not fully reach the recommended threshold of 0.90—potentially attributable to the large sample size and complexity of clinical data—the overall improvement in model fit provided a robust foundation for subsequent path analysis. Based on the final revised model, the reliability and validity of the measurement model were evaluated. As shown in Table 3, the standardized factor loadings for the suicidal ideation latent variable ranged from 0.736 to 0.743, with composite reliability (CR) of 0.705 and average variance extracted (AVE) of 0.547, indicating good convergent validity. Although the AVE values for childhood maltreatment and difficulties in emotion regulation were slightly below 0.50, their CR values reached 0.781 and 0.824, respectively, both substantially exceeding the recommended threshold of 0.70. Moreover, the observed dimensions possessed significant clinical content validity. Therefore, the construct validity of the measurement model was considered acceptable.

**Table 3:** Results of the measurement model reliability and validity analysis.

Latent construct	Indicator	λ	e	CR	AVE
Childhood maltreatment	Physical abuse	0.592	0.650	0.744	0.399
	Emotional abuse	0.887	0.213		
	Physical neglect	0.587	0.655		
	Emotional neglect	0.690	0.524		
	Sexual abuse	0.196	0.962		
Emotion dysregulation	Non-acceptance	0.58	0.664	0.800	0.443
	Goals	0.115	0.987		
	Impulsivity	0.9	0.190		
	Awareness	0.804	0.354		
	Strategy	0.45	0.798		
	Clarity	0.807	0.349		
Suicidal Ideation	suicidal ideation during the past week	0.736	0.458	0.707	0.547
	suicidal ideation during the most depressed period	0.743	0.448		

Path analysis revealed that, after controlling for relevant clinical and demographic variables, childhood maltreatment significantly and positively predicted difficulties in emotion regulation (*β* = 0.40, *p* < 0.001) and suicidal ideation (*β* = 0.23, *p* < 0.001). Difficulties in emotion regulation also significantly and positively predicted suicidal ideation (*β* = 0.53, *p* < 0.001). These findings preliminarily confirmed that difficulties in emotion regulation partially mediated the relationship between childhood maltreatment and suicidal ideation. Regarding the predictive effects of control variables, the model results (see Table 4) indicated that sex (*β* = 0.076, *p* = 0.038), family history (*β* = −0.082, *p* = 0.023), and age at first onset (*β* = −0.139, *p* = 0.004) significantly predicted suicidal ideation. Specifically, female sex, positive family history, and younger age at first onset were associated with higher levels of suicidal ideation. However, educational level, illness duration, and number of episodes/relapses did not reach statistical significance in predicting suicidal ideation (*p* > 0.05). After rigorously controlling for these significant demographic and clinical variables, the indirect pathway from childhood maltreatment to suicidal ideation through difficulties in emotion regulation remained highly significant (*p* < 0.001), providing robust evidence for the stability of this mediating mechanism across different clinical contexts.

**Table 4:** Intermediary model coefficient table.

Path (relationship)	β (std. coeff.)	S. E.	Z-value	*p*	95% BC Boot CI
Direct effects					
CT → DE	0.402	0.040	3.919	0.001	[0.323, 0.482]
DE → BS	0.532	0.049	7.382	< 0.001	[0.431, 0.625]
CT → BS	0.233	0.055	2.608	< 0.001	[0.121, 0.337]
Indirect effect					
CT → DE → BS	0.214	0.031	6.900	< 0.001	[0.159, 0.285]
Control variable					
Gender → BS	0.076	0.044	1.846	0.038	[0.008,0.208]
Family history → BS	−0.082	0.044	−2.015	0.023	[−0.284,-0.017]
Education level → BS	0.012	0.052	0.250	0.831	[−0.054,0.07]
Age of onset → BS	−0.139	0.055	−2.737	0.004	[−0.193,-0.041]
Duration of illness → BS	−0.022	0.048	−0.500	0.63	[−0.07,0.04]
Frequency of relapse → BS	−0.055	0.046	−1.321	0.122	[−0.103,0.01]

CT, Childhood maltreatment; DE, Emotion dysregulation; BS,Suicidal Ideation.

Furthermore, the bootstrap method was employed to test the mediating effect, with 2,000 resamples drawn from the original data (Table 5). Effects were considered statistically significant if the 95% bias-corrected confidence interval did not contain zero. The results revealed that the 95% confidence intervals for the total effect, direct effect, and indirect effect all excluded zero, indicating that difficulties in emotion regulation partially mediated the relationship between childhood maltreatment and suicidal ideation. The mediating effect of difficulties in emotion regulation accounted for 47.87% of the total effect.

**Table 5:** Bootstrap test of the mediating effect of difficulties in emotion regulation.

Path type	Standardized effect (β)	95% BC Boot CI (lower)	95% BC Boot CI (upper)	*p*	Effect size (%)
Total effect	0.447	0.358	0.528	< 0.001	—
Direct effect	0.233	0.121	0.337	< 0.001	52.13%
Indirect effect	0.214	0.159	0.285	< 0.001	47.87%

## 4 Discussion

This study found that childhood maltreatment was significantly associated with greater suicidal ideation in patients with depression. Specifically, emotional abuse, emotional neglect, physical neglect, and physical abuse each showed a significant association with suicidal ideation, whereas sexual abuse was not significantly linked to it. Among these forms of maltreatment, emotional abuse demonstrated the strongest association with suicidal ideation, followed by emotional neglect.

These findings support Hypothesis 1, indicating that childhood maltreatment is significantly associated with increased suicidal ideation. This result aligns with prior research conducted in patients with bipolar disorder (Laghaei et al., 2023). From a psychodynamic perspective, childhood abuse may evoke feelings of guilt and shame, which in turn are associated with undermine self-esteem and stress regulation in adulthood, thereby contributing to depressive affect and suicidal ideation. Prior studies have also documented a robust association between emotional abuse and suicidal ideation (Schønning et al., 2022; Peng et al., 2024). Peng et al. (2024) argued that emotional abuse in childhood conveys harmful beliefs about the self; when such beliefs are internalized over time, they form negative and hopeless cognitive schemas that increase the likelihood of suicidal ideation. Neuroimaging work by van Harmelen and others has shown that emotional abuse is associated with reduced activation in the medial prefrontal cortex, a region implicated in cognitive control and self-referential processing, which may be related to suicidal behavior (Carvalho Fernando et al., 2014; Üçok et al., 2015).

In the context of emotional neglect (van Harmelen et al., 2014), parental failure to respond to children’s emotional needs may lead to low mood, difficulties in emotion regulation and, ultimately, anhedonia and suicidal ideation. Individuals who have experienced physical neglect (Sekowski et al., 2020) may develop negative emotions because caregivers fail to provide basic care and support, thereby increasing the risk of suicidal ideation. Although physical abuse was significantly associated with suicidal ideation in this study, the magnitude of this association was modest. In the Chinese cultural context, traditional child-rearing practices have tended to be relatively strict. Although physical punishment has been increasingly challenged in recent years, milder forms of corporal discipline may still be implicitly accepted, which could attenuate the perceived psychological impact of physical abuse. With regard to sexual abuse, no significant association with suicidal ideation was found. This may be related to sample heterogeneity and limited statistical power. Moreover, sexual abuse is often accompanied by intense feelings of shame and stigma, which can make individuals reluctant to disclose such experiences (Briere et al., 2016; Yoon et al., 2018; Liu et al., 2022). Overall, these findings underscore the importance of systematically assessing childhood experiences in patients with depression as an integral component of a comprehensive evaluation for suicidal ideation, aiding in the identification of patients at heightened risk for suicidal ideation.

The results further showed that emotion dysregulation was associated with the relationship between childhood maltreatment and suicidal ideation in patients with depression. The pattern of associations suggests that childhood maltreatment may be related to suicidal ideation indirectly through its association with impaired emotion regulation capacities, while emotion dysregulation represented a more proximal factor linking early adversity to suicidal ideation. Previous studies (Nock, 2009; Bentley et al., 2014) have demonstrated that emotion dysregulation is a powerful mechanism connecting childhood maltreatment with self-injurious behavior in adolescence, thereby supporting Hypothesis 2 within the limits of cross-sectional data. Linehan’s biosocial model (Linehan, 1993) posits that the interaction between emotional vulnerability and invalidating or threatening environments contributes to deficits in emotion regulation skills, which are associated with suicidal behavior. Healthy emotion regulation involves several core components, including the ability to identify emotions, tolerate distress and use adaptive regulation strategies (Berking and Lukas, 2015). These skills typically emerge in the context of early interpersonal emotional exchanges. Exposure to abuse or neglect in childhood may disrupt the development of these skills, leaving individuals unable to manage emotional responses effectively and thereby increasing vulnerability to clinically significant depression (Alink et al., 2009; Khosravani et al., 2021). When confronted with stress, patients with depression often lack effective strategies to regulate negative emotions and tend to rely on maladaptive emotion regulation patterns (Raes and Hermans, 2008; Hopfinger et al., 2016).

Consistent with this view, the present study found that limited access to adaptive regulation strategies and impulse control difficulties were strongly associated with suicidal ideation. Maladaptive regulation strategies may lead individuals to cope with stress through avoidance or other passive responses, while impaired impulse control increases the likelihood of engaging in extreme behaviors. Together, these vulnerabilities are associated with heightened risk of developing suicidal ideation and, in some cases, with progression to suicidal behavior. These findings highlight the critical role of emotion regulation in understanding suicidal ideation. Clinical practice should therefore place particular emphasis on strengthening emotion regulation abilities in patients with depression, for example, by helping them expand their repertoire of adaptive strategies—which may contribute to alleviating depressive symptoms and suicidal ideation, potentially reducing suicide risk and improving clinical outcomes.

Taken together, the findings suggest that patients with depression who have experienced childhood maltreatment may be prone to intense and persistent suicidal ideation, and that emotion dysregulation further may further exacerbate this tendency. Future research with longitudinal designs is needed to determine whether interventions that specifically target emotion dysregulation could be especially important for reducing suicidal ideation in this population. In developing psychological treatment plans for patients with depression, routine assessment of childhood maltreatment history and emotion regulation difficulties is warranted. Group-based interventions that focus on enhancing positive emotion regulation and emotional understanding may be beneficial. In addition, trauma-focused cognitive–behavioral approaches may help patients process and accept traumatic childhood experiences and their associated emotions, thereby potentially improving emotion regulation capacities. Future research should prioritize the development and evaluation of such interventions with the aim of reducing suicidal ideation and suicide rates among clinically depressed patients.

This study has several limitations. First, the cross-sectional design precludes causal inferences; prospective longitudinal studies are needed to confirm and extend the present findings. Second, reliance on self-report and retrospective assessment may have introduced inaccuracies in measurement of key variables. Third, data were obtained from a single clinical setting, which may limit the generalizability and robustness of the results. Future work should adopt multicenter designs and larger samples to replicate and validate these findings. Overall, fostering healthy emotion regulation skills may be crucial for patients with depression who experience suicidal ideation, while preventing childhood maltreatment remains fundamental for reducing lifelong adverse consequences.

## 5 Conclusion

1. Childhood maltreatment was identified as a correlate of suicidal ideation in patients with depression. Specifically, with the exception of sexual abuse, the subtypes of emotional abuse, emotional neglect, physical abuse, and physical neglect were each consistently associated with increased suicidal ideation. Furthermore, a dose–response relationship was observed, wherein greater severity of maltreatment was associated with stronger suicidal ideation.
2. In this clinical sample of patients with depression, emotion dysregulation was significantly associated with greater suicidal ideation. Specifically, all its dimensions—non-acceptance of emotional responses, difficulties in emotional awareness, limited access to emotion regulation strategies, impulse control difficulties, difficulties in emotional understanding, and difficulties in goal-directed behavior—were each significantly associated with individual levels of suicidal ideation.
3. Statistical mediation analysis indicated that emotion dysregulation played a mediating role in the association between childhood maltreatment and suicidal ideation within the limits of this cross-sectional design. More severe childhood maltreatment was associated with poorer emotion regulation capacities, which in turn were associated with more severe suicidal ideation.

## Data Availability

The original contributions presented in the study are included in the article/supplementary material, further inquiries can be directed to the corresponding author.
